# Cubic ZrW_1.75_Mo_0.25_O_8_ from a Rietveld refinement based on neutron powder diffraction data

**DOI:** 10.1107/S1600536809015281

**Published:** 2009-04-30

**Authors:** Xuebin Deng, Yilong Cao, Juzhou Tao, Xinhua Zhao

**Affiliations:** a College of Chemistry, Beijing Normal University, Beijing 100875, People’s Republic of China; b Experimental Physics Center, Institute of High Energy Physics, Chinese Academy of Sciences, Beijing 100049, People’s Republic of China

## Abstract

The solid solution in the system Zr–Mo–W–O with composition ZrW_1.75_Mo_0.25_O_8_ (zirconium tungsten molybdenum octa­oxide) was prepared by solid-state reactions as a polycrystalline material. Its structure has cubic symmetry (space group *P*2_1_3) at room temperature. The structure contains a network of corner-sharing ZrO_6_ octa­hedra (.3. symmetry) and *M*O_4_ (*M* = W, Mo) tetra­hedra (.3. symmetry). Along the main threefold axis of the cubic unit cell, the *M*O_4_ tetra­hedra are arranged in pairs forming *M*_2_O_8_ units in which the *M*1O_4_ tetra­hedra have larger distortions in terms of bond distances and angles than the *M*2O_4_ tetra­hedra. These units are disordered over two possible orientations, with the *M*—O_terminal_ vectors pointing to the [111] or [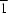 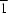 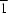] directions. The reversal of the orientations of the *M*_2_O_8_ units results from the concerted flips of these units. The time-averaged proportions of flipped and unflipped *M*_2_O_8_ units were determined and the fraction of unflipped *M*_2_O_8_ units is about 0.95. The order degree of the *M*_2_O_8_ unit orientation is about 0.9. During the reversal process, the *M*-atom site has a migration about 0.93 Å, one of the O-atom sites has a 0.25 Å migration distance, whereas two other O-atom sites migrate marginally (≃ 0.08 Å). The results prove the constraint strategy to be a reasonable approach based on the ratcheting mechanism.

## Related literature

For a general description of the structures and properties of unsubstituted ZrW_2_O_8_, see: Mary *et al.* (1996 ▶); Evans *et al.* (1996 ▶, 1999 ▶). Details on the ratcheting mechanism have been described by Hampson *et al.* (2004 ▶, 2005 ▶). For the synthesis of the title compound, see: Zhao *et al.* (2007 ▶). For a detailed description of polyhedral distortion parameters, see: Baur (1974 ▶); Robinson *et al.* (1971 ▶). For isomorphism and polymorphism in cubic ZrW_2_O_8_ type compounds, see: Evans *et al.* (2000 ▶); Lind *et al.* (1998 ▶); Huang *et al.*(2005 ▶); Deng *et al.* (2008 ▶). For their unusual isotropic negative thermal expansion properties, see: Mary *et al.* (1996 ▶). 

## Experimental

### 

#### Crystal data

ZrW_1.75_Mo_0.25_O_8_
*M* *_r_* = 564.93
Cubic, 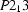

*a* = 9.156880 (17) Å
*V* = 767.79 (1) Å^3^
*Z* = 4
Time-of-flight radiation
λ = 0.5–4.4 Å
*T* = 298 K
Specimen shape: cylinder
30 × 10 × 10 mm

#### Data collection

GPPD diffractometer
Specimen mounting: standard cylindrical vanadium sample holder
Scan method: time of flight
2θ_min_ = 53, 2θ_max_ = 145°

#### Refinement

*R*_p_ = 0.032
*R*_wp_ = 0.044
1893 reflections
58 parameters

### 

Data collection: IPNS local software (Worlton *et al.*, 2006 ▶); cell refinement: *GSAS* (Larson & von Dreele (2000 ▶); data reduction: IPNS local software; program(s) used to solve structure: *GSAS*; program(s) used to refine structure: *GSAS*; molecular graphics: *VICS-II* (Izumi & Dilanian, 2005 ▶); software used to prepare material for publication: *GSAS*.

## Supplementary Material

Crystal structure: contains datablocks global, I, DXB-W1.75_p_01, DXB-W1.75_p_02. DOI: 10.1107/S1600536809015281/wm2222sup1.cif

Rietveld powder data: contains datablocks I, I, I. DOI: 10.1107/S1600536809015281/wm2222Isup2.rtv

Additional supplementary materials:  crystallographic information; 3D view; checkCIF report

## Figures and Tables

**Table:** 

Zr1—O1^i^	2.0384 (21)
Zr1—O2^ii^	2.0915 (20)
Zr1—O1-1^ii^	2.0261 (21)
Zr1—O2-1^i^	2.1037 (19)
*M*1—O1	1.8069 (18)
*M*1—O3	2.405 (4)
*M*1—O4	1.7105 (32)
*M*1—*M*2-1	0.9294 (23)
*M*2—O2	1.7933 (16)
*M*2—O3	1.729 (4)
O1—O2-1	0.084 (4)
O3—O3-1	0.254 (7)

**Table:** 

O1—*M*1—O1^iii^	115.82 (6)
O1—*M*1—O4	101.96 (9)
O2—*M*2—O2^iii^	109.66 (9)
O2—*M*2—O3	109.28 (9)

*M* = Mo, W. Symmetry codes: (i) 
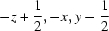
; (ii) 
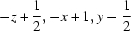
; (iii) 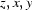.

**Fig. 1.**
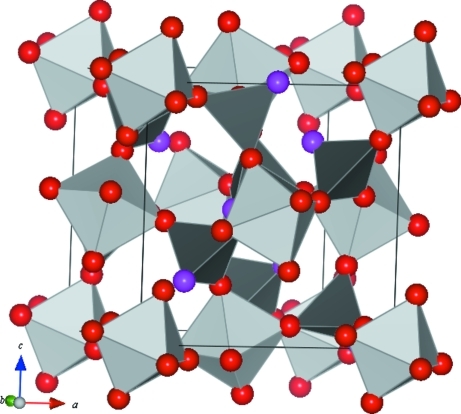
Polyhedral representation of cubic ZrW1.75Mo0.25O8. ZrO6 octahedra and MO4 tetrahedra are shown as light-gray and dark-gray polyhedra. The bridging oxygen atoms and the terminal oxygen atoms are shown as red and pink balls, respectively. For clarity, the atoms related to the flipped part are removed from the figure.

**Fig. 2.**
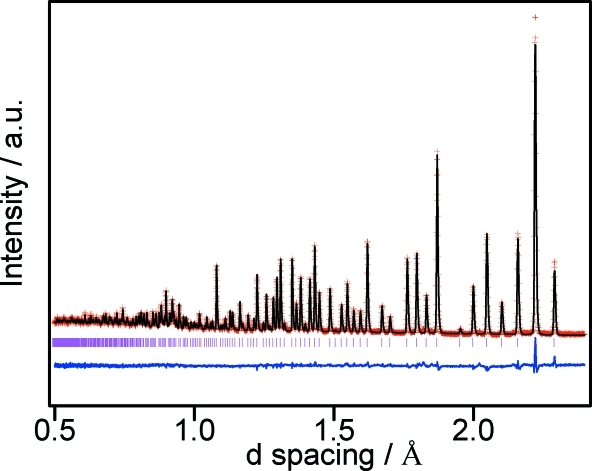
Final Rietveld refinement neutron powder data plot and difference plot for cubic ZrW1.75Mo0.25O8. The crosses are the observed data, the solid line is the calculated pattern, and the tick marks indicate the calculated reflection positions. The difference curve is plotted below. Only the 145° bank data is shown.

**Fig. 3.**
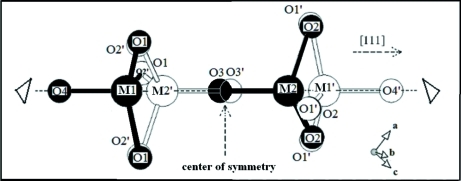
The schematic representation of the starting structural model for cubic ZrW1.75Mo0.25O8. Black balls and circles represent 'unflipped' and 'flipped' atoms, respectively.

## supplementary crystallographic information

### Comment

Cubic ZrW_2_O_8_ type compounds have been attracting considerable interests due to their unusual isotropic negative thermal expansion properties (Mary *et al.*, 1996). Many research groups have been studying isomorphism and polymorphism behaviors of this family (Evans *et al.*, 2000; Deng *et al.*, 2008; Huang *et al.*, 2005; Lind *et al.*,1998), to investigate the structure-property relationships in detail, and to explore procedures for improving the properties of such materials. By means of Mo atoms partially substituting W atoms to form a cubic solid solution of the type ZrW_2 - _*_x_*Mo*_x_*O_8_, we were able to extend the thermal stability range of cubic ZrW_2_O_8_ type compounds. Recently, we have reported the high-temperature synthesis of the solid solution ZrW_2 - _*_x_*Mo*_x_*O_8_ series (Zhao *et al.*, 2007), and found that the order-disorder phase transition temperature (T_tr_) and the order degree of the ZrW_2 - _*_x_*Mo*_x_*O_8_ series decrease with the increase of the Mo concentration. According to the X-ray diffraction patterns, these compounds (*x* < 0.9) adopt the ordered α-ZrW_2_O_8 _structure at room temperature. However, the detailed average crystal structures are not reported.

In this paper, we describe the synthesis of cubic ZrW_1.75_Mo_0.25_O_8_ and the average crystal structure as determined from neutron powder diffraction data using the Rietveld method. The crystal structure is presented in Fig.1 and plot of the Rietveld refinement is shown in Fig. 2.

The structure of ZrW_1.75_Mo_0.25_O_8_ contains a network of corner-sharing ZrO_6_ octahedra and *M*O_4_ tetrahedra. Each octahedron shares all six corners with six separate *M*O_4_ tetrahedra, but each *M*O_4_ tetrahedron shares only three of its four oxygen corners with adjacent octahedra. In other words, each *M*O_4_ tetrahedron has a 'terminal' oxygen atom. Along the main 3-fold axis of the cubic unit cell, the *M*O_4_ tetrahedra are arranged in pairs forming condensed *M*_2_O_8_ units. The 'terminal' O atom of each *M*O_4_ tetrahedron points in the same direction in the *M*_2_O_8_ unit, leading to an O4-*M*1···O3-*M*2 arrangement (Fig. 3). The *M*-O_terminal_ bond lengths are significantly shorter than the other *M*-O bond lengths. The second nearest *M* atom to terminal O4 atom lies at a distance of about 3.6 Å, so O4 atom is strictly one-coordinate. However, the distance to the terminal O3 atom is about 2.4 Å, indicating that it has one short and one long bond to the *M* atom. The existence of some interaction between *M*1 and the terminal O3 atom of the adjacent *M*2O_4_ tetrahedron is proved by larger distortions in terms of bond angles and distances of *M*1O_4_ tetrahedra than that of *M*2O_4_ tetrahedra. The values of the bond length distortion index D (Baur, 1974), 0.02028, and of the bond angle variance σ^2^ (Robinson *et al.*, 1971), 58.12 °^2^, of *M*1O_4_ tetrahedra are both larger than that of *M*2O_4_ tetrahedra in which D is 0.01355 and σ^2^ is 0.0445. Moreover, the O1—O3 distance of 2.69Å is shorter than all other O—O distances, indicating there is a weak repulsive interaction between these two atoms. This is supported by bond angle values; O1—*M*1—O1 is larger than O2—*M*2—O2 and O1—*M*1—O4 is smaller than O2—*M*2—O3.

Based on the structure model of α-ZrW_2_O_8_ (Evans *et al.*, 1996, 1999), some special conditions were constrained to match the 'ratcheting mechanism' described by Hampson *et al.* (2004, 2005) that considers the static (averaged) crystal structure determined by X-ray diffraction and the NMR observation of oxygen dynamics. The refinement results indicate that before and after the reversal process, the *M* atom site has a migration about a 0.93 Å, the O3 atom site has a 0.25Å migration, whereas the O1/O2 atom site migrate only a little (0.08 Å). The time-averaged proportion of flipped and unflipped *M*_2_O_8_ units was determined. The fraction of unflipped *M*_2_O_8_ units is about 0.95 and the order degree (defined as 2*f*-1, in which *f* is the site occupancy value of the unflipped part) is about 0.9.

### Experimental

Cubic ZrW_1.75_Mo_0.25_O_8_ was prepared using a similar procedure described previously (Zhao *et al.*, 2007). Analytical-grade reagents ZrOCl_2_^.^8H_2_O, (NH_4_)_10_W_12_O_41_^.^5H_2_O and (NH_4_)_6_Mo_7_O_24_^.^4H_2_O were dissolved in water and the solutions of Zr(IV) and Mo(VI) were simultaneously dropped into the slurry of the W(VI) compound to obtain a white precipitate. The precipitate was dried together with the mother liquor at 373 K and subsequently ground in an agate mortar to obtain a homogeneous powder. Then the powder was sintered at 873 K for 3 h. and then pressed into a pellet under a pressure of 4 MPa. Then the pellet was again sintered at 1403 K for 1 h and then quenched to room temperature to yield cubic ZrW_1.7_Mo_0.3_O_8_. During the sintering process, the weight loss of this sample, which is attributed to the volatility of MoO_3_, is 1.4 wt %, so the exact formula is ZrW_1.75_Mo_0.25_O_8_. The trace of ZrO_2_ as an impurity in the powder is undetectable by neutron diffraction.

### Refinement

The starting structure model for refinement is based on the crystal structure of α-ZrW_2_O_8_ (Evans *et al.* 1999). The schematic representation of the starting structure model is shown in Fig. 3. During the refinement sets of constraints of structure parameters were used to match the 'ratcheting mechanism' (Hampson *et al.*, 2004, 2005). The W sites of the original model are statistically occupied by W and Mo atoms (denoted with *M*). The corresponding atomic coordinates of flipped and unflipped *M*_2_O_8_ units are set to be centrosymmetric through the imaginary inversion center of the unit cell. The corresponding anisotropic atomic displacement parameters are constrained to be equal for each site. The initial occupancy fractions of the original atomic sites were set to 1 (the occupancy fraction sum of *M*1 and *M*2 were set to unity) while those of the derived atomic sites were set to 0. During the refinement process, the occupancy fraction of the atoms as a parameter in either unflipped or flipped part is constrained to maintain the chemical composition. Two histograms (bank 1 and bank 5) of data from GPPD were used in the refinement. The given reliability factors (see Tables) are the result of the combined refinement based on bank 1 and bank 5 data.

### Crystal data

**Table:**

ZrW_1.75_Mo_0.25_O_8_	*D*_x_ = 4.887 Mg m^−^^3^
*M**_r_* = 564.93	Time-of-flight radiation
Cubic, *P*2_1_3	*T* = 298 K
*a* = 9.156880 (17) Å	white
*V* = 767.79 (1) Å^3^	cylinder, 30 × 10 mm
*Z* = 4	

### Data collection

**Table:**

GPPD diffractometer	Scan method: time of flight
Specimen mounting: standard cylindrical vanadium sample holder	

### Refinement

**Table:**

Least-squares matrix: full	? data points
*R*_p_ = 0.032	58 parameters
*R*_wp_ = 0.044	55 constraints
*R*_exp_ = ?	(Δ/σ)_max_ = 0.02
χ^2^ = 2.341	

### Fractional atomic coordinates and isotropic or equivalent isotropic displacement parameters (Å^2^)

**Table:**

	*x*	*y*	*z*	*U*_iso_*/*U*_eq_	Occ. (<1)
Zr1	0.00068 (14)	0.00068 (14)	0.00068 (14)	0.01044	
W1	0.34038 (14)	0.34038 (14)	0.34038 (14)	0.01449	0.8339 (22)
W2	0.60102 (13)	0.60102 (13)	0.60102 (13)	0.01048	0.8339 (22)
O1	0.20648 (18)	0.4373 (2)	0.4482 (2)	0.02634	0.9530 (26)
O2	0.78912 (16)	0.5691 (2)	0.55685 (18)	0.01893	0.9530 (26)
O3	0.4920 (2)	0.4920 (2)	0.4920 (2)	0.02679	0.9530 (26)
O4	0.23253 (13)	0.23253 (13)	0.23253 (13)	0.03851	0.9530 (26)
Mo1	0.34038 (14)	0.34038 (14)	0.34038 (14)	0.01449	0.11913 (32)
Mo2	0.60102 (13)	0.60102 (13)	0.60102 (13)	0.01048	0.11913 (32)
W1-1	0.65963 (14)	0.65963 (14)	0.65963 (14)	0.01449	0.0411 (22)
W2-1	0.39898 (13)	0.39898 (13)	0.39898 (13)	0.01048	0.0411 (22)
Mo1-1	0.65963 (14)	0.65963 (14)	0.65963 (14)	0.01449	0.00587 (32)
Mo2-1	0.39898 (13)	0.39898 (13)	0.39898 (13)	0.01048	0.00587 (32)
O3-1	0.5080 (2)	0.5080 (2)	0.5080 (2)	0.02679	0.0470 (26)
O4-1	0.76747 (13)	0.76747 (13)	0.76747 (13)	0.03851	0.0470 (26)
O1-1	0.79352 (18)	0.5627 (2)	0.5518 (2)	0.02634	0.0470 (26)
O2-1	0.21088 (16)	0.4309 (2)	0.44315 (18)	0.01893	0.0470 (26)

### Atomic displacement parameters (Å^2^)

**Table:**

	*U*^11^	*U*^22^	*U*^33^	*U*^12^	*U*^13^	*U*^23^
Zr1	0.0104 (2)	0.0104 (2)	0.0104 (2)	−0.0002 (3)	−0.0002 (3)	−0.0002 (3)
W1	0.0145 (5)	0.0145 (5)	0.0145 (5)	0.0080 (6)	0.0080 (6)	0.0080 (6)
W2	0.0105 (5)	0.0105 (5)	0.0105 (5)	0.0006 (4)	0.0006 (4)	0.0006 (4)
O1	0.0198 (9)	0.0272 (12)	0.0320 (11)	0.0083 (8)	0.0142 (8)	−0.0013 (8)
O2	0.0054 (7)	0.0272 (10)	0.0242 (10)	0.0057 (6)	0.0016 (7)	0.0058 (7)
O3	0.0268 (5)	0.0268 (5)	0.0268 (5)	−0.0090 (6)	−0.0090 (6)	−0.0090 (6)
O4	0.0385 (6)	0.0385 (6)	0.0385 (6)	−0.0119 (6)	−0.0119 (6)	−0.0119 (6)
Mo1	0.0145 (5)	0.0145 (5)	0.0145 (5)	0.0080 (6)	0.0080 (6)	0.0080 (6)
Mo2	0.0105 (5)	0.0105 (5)	0.0105 (5)	0.0006 (4)	0.0006 (4)	0.0006 (4)
W1-1	0.0145 (5)	0.0145 (5)	0.0145 (5)	0.0080 (6)	0.0080 (6)	0.0080 (6)
W2-1	0.0105 (5)	0.0105 (5)	0.0105 (5)	0.0006 (4)	0.0006 (4)	0.0006 (4)
Mo1-1	0.0145 (5)	0.0145 (5)	0.0145 (5)	0.0080 (6)	0.0080 (6)	0.0080 (6)
Mo2-1	0.0105 (5)	0.0105 (5)	0.0105 (5)	0.0006 (4)	0.0006 (4)	0.0006 (4)
O3-1	0.0268 (5)	0.0268 (5)	0.0268 (5)	−0.0090 (6)	−0.0090 (6)	−0.0090 (6)
O4-1	0.0385 (6)	0.0385 (6)	0.0385 (6)	−0.0119 (6)	−0.0119 (6)	−0.0119 (6)
O1-1	0.0198 (9)	0.0272 (12)	0.0320 (11)	0.0083 (8)	0.0142 (8)	−0.0013 (8)
O2-1	0.0054 (7)	0.0272 (10)	0.0242 (10)	0.0057 (6)	0.0016 (7)	0.0058 (7)

### Geometric parameters (Å, °)

**Table:**

Zr1—O1^i^	2.0384 (21)	O3—M2	1.729 (4)
Zr1—O1^ii^	2.0384 (21)	O3—M1-1	2.659 (4)
Zr1—O1^iii^	2.0384 (21)	O3—M2-1	1.475 (4)
Zr1—O2^iv^	2.0915 (20)	O3—O3-1	0.254 (7)
Zr1—O2^v^	2.0915 (20)	O4—M1	1.7105 (32)
Zr1—O2^vi^	2.0915 (20)	O4—M2-1	2.6400 (29)
Zr1—O1-1^iv^	2.0261 (21)	M1-1—M2	0.9295 (23)
Zr1—O1-1^v^	2.0261 (21)	M1-1—O2	1.7261 (18)
Zr1—O1-1^vi^	2.0261 (21)	M1-1—O2^vii^	1.7261 (18)
Zr1—O2-1^i^	2.1037 (19)	M1-1—O2^viii^	1.7261 (18)
Zr1—O2-1^ii^	2.1037 (19)	M1-1—O3	2.659 (4)
Zr1—O2-1^iii^	2.1037 (19)	M1-1—O3-1	2.405 (4)
M1—O1	1.8069 (18)	M1-1—O4-1	1.7105 (32)
M1—O1^vii^	1.8069 (18)	M1-1—O1-1	1.8069 (18)
M1—O1^viii^	1.8069 (18)	M1-1—O1-1^vii^	1.8069 (18)
M1—O3	2.405 (4)	M1-1—O1-1^viii^	1.8069 (18)
M1—O4	1.7105 (32)	M2-1—M1	0.9294 (23)
M1—M2-1	0.9294 (23)	M2-1—M2	3.204 (4)
M1—O3-1	2.659 (4)	M2-1—O1	1.8528 (18)
M1—O2-1	1.7260 (18)	M2-1—O1^vii^	1.8528 (18)
M1—O2-1^vii^	1.7260 (18)	M2-1—O1^viii^	1.8528 (18)
M1—O2-1^viii^	1.7260 (18)	M2-1—O3	1.475 (4)
M2—O2	1.7933 (16)	M2-1—O4	2.6400 (29)
M2—O2^vii^	1.7933 (16)	M2-1—O3-1	1.729 (4)
M2—O2^viii^	1.7933 (16)	M2-1—O2-1	1.7933 (16)
M2—O3	1.729 (4)	M2-1—O2-1^vii^	1.7933 (16)
M2—M1-1	0.9295 (23)	M2-1—O2-1^viii^	1.7933 (16)
M2—M2-1	3.204 (4)	O3-1—M1	2.659 (4)
M2—O3-1	1.475 (4)	O3-1—M2	1.475 (4)
M2—O4-1	2.6399 (29)	O3-1—O3	0.254 (7)
M2—O1-1	1.8528 (18)	O3-1—M1-1	2.405 (4)
M2—O1-1^vii^	1.8528 (18)	O3-1—M2-1	1.729 (4)
M2—O1-1^viii^	1.8528 (18)	O4-1—M2	2.6399 (29)
O1—Zr1^ix^	2.0384 (21)	O4-1—M1-1	1.7105 (32)
O1—M1	1.8069 (18)	O1-1—Zr1^x^	2.0261 (21)
O1—M2-1	1.8528 (18)	O1-1—M2	1.8528 (18)
O1—O2-1	0.084 (4)	O1-1—O2	0.084 (4)
O2—Zr1^x^	2.0915 (20)	O1-1—M1-1	1.8069 (18)
O2—M2	1.7933 (16)	O2-1—Zr1^ix^	2.1037 (19)
O2—M1-1	1.7261 (18)	O2-1—M1	1.7260 (18)
O2—O1-1	0.084 (4)	O2-1—O1	0.084 (4)
O3—M1	2.405 (4)	O2-1—M2-1	1.7933 (16)
			
O1^i^—Zr1—O1^ii^	90.82 (10)	O2^viii^—M2—O3	109.28 (9)
O1^i^—Zr1—O1^iii^	90.82 (10)	O2^viii^—M2—M1-1	70.72 (9)
O1^i^—Zr1—O2^iv^	178.37 (10)	O2^viii^—M2—O3-1	109.28 (9)
O1^i^—Zr1—O2^v^	87.64 (8)	O2^viii^—M2—O1-1	110.24 (11)
O1^i^—Zr1—O2^vi^	89.74 (8)	O2^viii^—M2—O1-1^vii^	110.90 (10)
O1^i^—Zr1—O1-1^iv^	179.4968 (9)	O2^viii^—M2—O1-1^viii^	1.88 (12)
O1^i^—Zr1—O1-1^v^	88.83 (7)	O3—M2—M1-1	180.0
O1^i^—Zr1—O1-1^vi^	88.83 (7)	O3—M2—O1-1	107.43 (8)
O1^i^—Zr1—O2-1^ii^	92.01 (12)	O3—M2—O1-1^vii^	107.43 (8)
O1^i^—Zr1—O2-1^iii^	89.92 (10)	O3—M2—O1-1^viii^	107.43 (8)
O1^ii^—Zr1—O1^iii^	90.82 (10)	M1-1—M2—O3-1	180.0
O1^ii^—Zr1—O2^iv^	89.74 (8)	M1-1—M2—O1-1	72.57 (8)
O1^ii^—Zr1—O2^v^	178.37 (10)	M1-1—M2—O1-1^vii^	72.57 (8)
O1^ii^—Zr1—O2^vi^	87.64 (8)	M1-1—M2—O1-1^viii^	72.57 (8)
O1^ii^—Zr1—O1-1^iv^	88.83 (7)	O3-1—M2—O1-1	107.43 (8)
O1^ii^—Zr1—O1-1^v^	179.4968 (9)	O3-1—M2—O1-1^vii^	107.43 (8)
O1^ii^—Zr1—O1-1^vi^	88.83 (7)	O3-1—M2—O1-1^viii^	107.43 (8)
O1^ii^—Zr1—O2-1^i^	89.92 (10)	O1-1—M2—O1-1^vii^	111.43 (8)
O1^ii^—Zr1—O2-1^iii^	92.01 (12)	O1-1—M2—O1-1^viii^	111.43 (8)
O1^iii^—Zr1—O2^iv^	87.64 (8)	O1-1^vii^—M2—O1-1^viii^	111.43 (8)
O1^iii^—Zr1—O2^v^	89.74 (8)	Zr1^ix^—O1—M1	153.75 (12)
O1^iii^—Zr1—O2^vi^	178.37 (10)	Zr1^ix^—O1—M2-1	174.36 (12)
O1^iii^—Zr1—O1-1^iv^	88.83 (7)	Zr1^ix^—O1—O2-1	139.9 (22)
O1^iii^—Zr1—O1-1^v^	88.83 (7)	M1—O1—M2-1	29.39 (8)
O1^iii^—Zr1—O1-1^vi^	179.4984 (9)	M2-1—O1—O2-1	44.3 (21)
O1^iii^—Zr1—O2-1^i^	92.01 (12)	Zr1^x^—O2—M2	171.71 (13)
O1^iii^—Zr1—O2-1^ii^	89.92 (10)	Zr1^x^—O2—M1-1	155.98 (13)
O2^iv^—Zr1—O2^v^	91.81 (10)	M2—O2—O1-1	133.9 (22)
O2^iv^—Zr1—O2^vi^	91.81 (10)	M2—O2—M1-1	30.55 (8)
O2^iv^—Zr1—O1-1^v^	90.60 (12)	M1-1—O2—O1-1	162.9 (23)
O2^iv^—Zr1—O1-1^vi^	92.72 (10)	M2—O3—M2-1	179.972
O2^iv^—Zr1—O2-1^i^	179.5134 (9)	M2-1—O3—O3-1	179.972
O2^iv^—Zr1—O2-1^ii^	88.52 (6)	M2—M1-1—O2	78.73 (10)
O2^iv^—Zr1—O2-1^iii^	88.53 (6)	M2—M1-1—O2^vii^	78.73 (10)
O2^v^—Zr1—O2^vi^	91.81 (10)	M2—M1-1—O2^viii^	78.73 (10)
O2^v^—Zr1—O1-1^iv^	92.72 (10)	M2—M1-1—O4-1	179.972
O2^v^—Zr1—O1-1^vi^	90.60 (12)	M2—M1-1—O1-1	78.04 (9)
O2^v^—Zr1—O2-1^i^	88.53 (6)	M2—M1-1—O1-1^vii^	78.04 (9)
O2^v^—Zr1—O2-1^ii^	179.5134 (9)	M2—M1-1—O1-1^viii^	78.04 (9)
O2^v^—Zr1—O2-1^iii^	88.52 (6)	O2—M1-1—O2^vii^	116.27 (6)
O2^vi^—Zr1—O1-1^iv^	90.60 (12)	O2—M1-1—O2^viii^	116.27 (6)
O2^vi^—Zr1—O1-1^v^	92.72 (10)	O2—M1-1—O4-1	101.27 (10)
O2^vi^—Zr1—O2-1^i^	88.52 (6)	O2—M1-1—O1-1^vii^	115.68 (10)
O2^vi^—Zr1—O2-1^ii^	88.53 (6)	O2—M1-1—O1-1^viii^	116.42 (11)
O2^vi^—Zr1—O2-1^iii^	179.5122 (9)	O2^vii^—M1-1—O2^viii^	116.27 (6)
O1-1^iv^—Zr1—O1-1^v^	91.52 (10)	O2^vii^—M1-1—O4-1	101.27 (10)
O1-1^iv^—Zr1—O1-1^vi^	91.52 (10)	O2^vii^—M1-1—O1-1	116.42 (11)
O1-1^iv^—Zr1—O2-1^i^	178.51 (10)	O2^vii^—M1-1—O1-1^viii^	115.68 (10)
O1-1^iv^—Zr1—O2-1^ii^	87.63 (8)	O2^viii^—M1-1—O4-1	101.27 (10)
O1-1^iv^—Zr1—O2-1^iii^	89.73 (9)	O2^viii^—M1-1—O1-1	115.68 (10)
O1-1^v^—Zr1—O1-1^vi^	91.52 (10)	O2^viii^—M1-1—O1-1^vii^	116.42 (11)
O1-1^v^—Zr1—O2-1^i^	89.73 (9)	O4-1—M1-1—O1-1	101.96 (9)
O1-1^v^—Zr1—O2-1^ii^	178.51 (10)	O4-1—M1-1—O1-1^vii^	101.96 (9)
O1-1^v^—Zr1—O2-1^iii^	87.63 (8)	O4-1—M1-1—O1-1^viii^	101.96 (9)
O1-1^vi^—Zr1—O2-1^i^	87.63 (8)	O1-1—M1-1—O1-1^vii^	115.82 (6)
O1-1^vi^—Zr1—O2-1^ii^	89.73 (9)	O1-1—M1-1—O1-1^viii^	115.82 (6)
O1-1^vi^—Zr1—O2-1^iii^	178.51 (10)	O1-1^vii^—M1-1—O1-1^viii^	115.82 (6)
O2-1^i^—Zr1—O2-1^ii^	91.13 (9)	M1—M2-1—O1	72.57 (8)
O2-1^i^—Zr1—O2-1^iii^	91.13 (9)	M1—M2-1—O1^vii^	72.57 (8)
O2-1^ii^—Zr1—O2-1^iii^	91.13 (9)	M1—M2-1—O1^viii^	72.57 (8)
O1—M1—O1^vii^	115.82 (6)	M1—M2-1—O3	179.9802
O1—M1—O1^viii^	115.82 (6)	M1—M2-1—O3-1	179.972
O1—M1—O4	101.96 (9)	M1—M2-1—O2-1	70.72 (9)
O1—M1—M2-1	78.04 (9)	M1—M2-1—O2-1^vii^	70.72 (9)
O1—M1—O2-1^vii^	116.42 (11)	M1—M2-1—O2-1^viii^	70.72 (9)
O1—M1—O2-1^viii^	115.68 (10)	O1—M2-1—O1^vii^	111.43 (8)
O1^vii^—M1—O1^viii^	115.82 (6)	O1—M2-1—O1^viii^	111.43 (8)
O1^vii^—M1—O4	101.96 (9)	O1—M2-1—O3	107.43 (8)
O1^vii^—M1—M2-1	78.04 (9)	O1—M2-1—O3-1	107.43 (8)
O1^vii^—M1—O2-1	115.68 (10)	O1—M2-1—O2-1^vii^	110.90 (10)
O1^vii^—M1—O2-1^viii^	116.42 (11)	O1—M2-1—O2-1^viii^	110.24 (11)
O1^viii^—M1—O4	101.96 (9)	O1^vii^—M2-1—O1^viii^	111.43 (8)
O1^viii^—M1—M2-1	78.04 (9)	O1^vii^—M2-1—O3	107.43 (8)
O1^viii^—M1—O2-1	116.42 (11)	O1^vii^—M2-1—O3-1	107.43 (8)
O1^viii^—M1—O2-1^vii^	115.68 (10)	O1^vii^—M2-1—O2-1	110.24 (11)
O4—M1—M2-1	179.9802	O1^vii^—M2-1—O2-1^viii^	110.90 (10)
O4—M1—O2-1	101.27 (10)	O1^viii^—M2-1—O3	107.43 (8)
O4—M1—O2-1^vii^	101.27 (10)	O1^viii^—M2-1—O3-1	107.43 (8)
O4—M1—O2-1^viii^	101.27 (10)	O1^viii^—M2-1—O2-1	110.90 (10)
M2-1—M1—O2-1	78.73 (10)	O1^viii^—M2-1—O2-1^vii^	110.24 (11)
M2-1—M1—O2-1^vii^	78.73 (10)	O3—M2-1—O2-1	109.28 (9)
M2-1—M1—O2-1^viii^	78.73 (10)	O3—M2-1—O2-1^vii^	109.28 (9)
O2-1—M1—O2-1^vii^	116.28 (6)	O3—M2-1—O2-1^viii^	109.28 (9)
O2-1—M1—O2-1^viii^	116.28 (6)	O3-1—M2-1—O2-1	109.28 (9)
O2-1^vii^—M1—O2-1^viii^	116.28 (6)	O3-1—M2-1—O2-1^vii^	109.28 (9)
O2—M2—O2^vii^	109.66 (9)	O3-1—M2-1—O2-1^viii^	109.28 (9)
O2—M2—O2^viii^	109.66 (9)	O2-1—M2-1—O2-1^vii^	109.66 (9)
O2—M2—O3	109.28 (9)	O2-1—M2-1—O2-1^viii^	109.66 (9)
O2—M2—M1-1	70.72 (9)	O2-1^vii^—M2-1—O2-1^viii^	109.66 (9)
O2—M2—O3-1	109.28 (9)	M2—O3-1—O3	179.972
O2—M2—O1-1^vii^	110.24 (11)	M2—O3-1—M2-1	179.972
O2—M2—O1-1^viii^	110.90 (10)	Zr1^x^—O1-1—M2	174.64 (11)
O2^vii^—M2—O2^viii^	109.66 (9)	Zr1^x^—O1-1—O2	140.0 (22)
O2^vii^—M2—O3	109.28 (9)	Zr1^x^—O1-1—M1-1	153.95 (13)
O2^vii^—M2—M1-1	70.72 (9)	Zr1^ix^—O2-1—M1	155.76 (13)
O2^vii^—M2—O3-1	109.28 (9)	Zr1^ix^—O2-1—M2-1	171.56 (13)
O2^vii^—M2—O1-1	110.90 (10)	M1—O2-1—O1	162.9 (23)
O2^vii^—M2—O1-1^viii^	110.24 (11)	O1—O2-1—M2-1	133.9 (22)

Symmetry codes: (i) −*z*+1/2, −*x*, *y*−1/2; (ii) *y*−1/2, −*z*+1/2, −*x*; (iii) −*x*, *y*−1/2, −*z*+1/2; (iv) −*z*+1/2, −*x*+1, *y*−1/2; (v) *y*−1/2, −*z*+1/2, −*x*+1; (vi) −*x*+1, *y*−1/2, −*z*+1/2;  (vii) *z*, *x*, *y*; (viii) *y*, *z*, *x*; (ix) −*z*, *x*+1/2, −*y*+1/2; (x) −*z*+1, *x*+1/2, −*y*+1/2.
